# Public preferences for corporate social responsibility activities in the pharmaceutical industry: Empirical evidence from Korea

**DOI:** 10.1371/journal.pone.0221321

**Published:** 2019-08-20

**Authors:** Hankil Lee, Sang Yong Kim, Goun Kim, Hye-Young Kang

**Affiliations:** 1 College of Pharmacy, Yonsei Institute of Pharmaceutical Sciences, Yonsei University, Incheon, South Korea; 2 Division of Pharmaceutical Outcomes and Policy, UNC Eshelman School of Pharmacy, University of North Carolina at Chapel Hill, Chapel Hill, North Carolina, United States of America; 3 Korea University Business School, Seoul, South Korea; Universidad de Zaragoza Facultad de Empresa y Gestion Publica, SPAIN

## Abstract

Although corporate social responsibility (CSR) activities are common in the pharmaceutical industry, there is little empirical evidence on consumer responses to CSR practices. We investigated public awareness, preferences, and expectations regarding social contribution of the pharmaceutical industry’s CSR activities, and identified the factors associated with such activities. We conducted an online survey with 1,298 respondents comprising two groups: healthy individuals (546) and patients (752). Most respondents (78%) expressed interest in CSR activities undertaken by pharmaceutical companies. However, they reported a lack of awareness and experience thereof; only 26.9% were aware of and 7.9% had experience with such activities. Among our six CSR activity categories, both survey groups showed the highest preference for the “promoting public health” (healthy group: 6.34/10; patient group: 6.37/10) and “emergency disaster relief support” (6.31 and 6.35) categories. Among sub-categories, activities related to “development of innovative drugs in untreated areas” (6.63 and 6.82) and “support for research on new drug development” (6.59 and 6.84) received the highest scores. The mean expectation score of social contribution of all CSR activities was slightly higher than the mean preference score (6.37 and 6.06, respectively). The patient group exhibited a larger difference between the highest and lowest expectation scores than the healthy group (1.11 and 0.64, respectively). The results of the regression analysis revealed that being a patient, being male, and having positive attitudes toward CSR and its expected effects significantly and positively affected public preferences regarding CSR activities. We can conclude that CSR activities with high public preference might be an effective strategy to improve public awareness of the pharmaceutical industry’s CSR activities. Furthermore, the highest preference for CSR activities relates to new drug development, indicating that our society believes the pharmaceutical industry’s key CSR activity should be to pursue its intrinsic mission: to fulfill unmet medical needs by developing new drugs.

## Introduction

Corporate social responsibility (CSR) refers to business operations involving initiatives that benefit society [1]. It has been adopted in many business sectors. CSR is also characterized as a business approach that aims sustainable development by providing economic, social, and environmental benefits to all stakeholders [2]. A business’ CSR activities can encompass a wide variety of tactics, such as donating to national and local charities (philanthropy), doing good deeds without expecting anything in return (volunteering), treating employees fairly and ethically (ethical labor practices), and implementing greener business operations (environmental efforts) [3, 4]. CSR activities may improve a firm’s reputation by differentiating it from rivals, thus providing a competitive advantage [5, 6]. Firms today realize that their activities can affect not only their own stakeholders but also the environment and society. As a result, CSR has become a part of core business operations which create shared value for businesses and society [7].

To effectively carry out CSR activities, one must understand consumers’ perceptions of CSR activities and identify CSR items to which consumers are most likely to respond [8]. A study by Schmeltz showed that consumers are more interested in CSR activities than companies expect and that there is a need for a balance between what companies can offer and what consumers want [9]. Schmeltz also reported that consumer-oriented CSR communication is crucial in overcoming consumer skepticism towards and disbelief in a company’s CSR activities. Thus, for CSR activities to have a more positive impact, it is necessary to investigate consumer perception of firms that engage in CSR and consumers’ preferred CSR activities, while considering the characteristics of each industry. In the pharmaceutical industry, consumers cannot directly purchase certain drugs, which could discourage pharmaceutical companies’ from conducting CSR activities. For example, to purchase prescription drugs, consumers must have a prescription from a physician, and in Korea, prescription drugs cannot be directly marketed to consumers [10]. Consumer preferences for and perceptions of CSR activities in the pharmaceutical industry differ from that in other industries due to the pharmaceutical industry’s structural characteristics. For example, the food industry is highly dependent on the economy; it is characterized by its consumption of raw materials or the natural environment. Consumers also clearly know what they are eating, so CSR activities in the food industry need to be differentiated according to different consumer preferences [11]. In the food industry, CSR activities related to environmental responsibility and animal welfare reportedly increase consumers’ marginal willingness to pay for food [12, 13]. McWilliams and Siegel observed that strategic implementation of CSR practices to meet consumer preferences (e.g., emphasizing that a hybrid car produces fewer pollutants, supporting animal welfare, or promoting organic foods) was a key business strategy for all industries [14]. In addition, since consumers are often influenced by the seller’s credibility when purchasing experimental products such as automobiles, appliances, and mutual funds, companies that sell those products tend to more actively engage in CSR activities than those that sell search goods such as clothing, shoes, and furniture [15].

As in other industries, the importance of CSR is well-recognized in the pharmaceutical industry, and it is a common practice therein. However, empirical evidence regarding the public perception of or response to CSR activities of pharmaceutical companies is sparse when compared to that in other business sectors. Only a few studies have explored CSR behavior among pharmaceutical companies. Droppert and Bennett investigated the types of CSR activities pursued by the pharmaceutical industry and the motivations for CSR, through in-depth interviews with representatives from a pharmaceutical company [16]. Another study that conducted a survey with medical and pharmacy students regarding their perception of CSR activities by the pharmaceutical industry showed that the respondents acknowledged the need for and the positive effects of CSR activities, while they also thought that some CSR activities should be abandoned [17]. CSR in the pharmaceutical industry is often recommended by governments and international organizations. For example, the UK Department for International Development and the United Nations have recommended a list of CSR actions to pharmaceutical companies to improve public access to essential medicines, especially in low- and middle-income countries, and to develop medicines for neglected diseases, the UK Department for International Development and the United Nations have recommended a list of CSR actions to pharmaceutical companies [18–21]. Therefore, the need for CSR in the pharmaceutical industry is increasing; however, prior studies focused on representatives of the pharmaceutical industry or future healthcare providers while neglecting to understand public perception and behavior regarding CSR.

Like in other developed countries and mature economic markets, CSR practices by pharmaceutical companies are common in Korea. Based on publicly available data from company websites and annual reports, we find a variety of CSR activities practiced by both international and domestic pharmaceutical companies in Korea (S1 Table). However, as in other parts of the world, there is a paucity of empirical research regarding consumer responses to CSR practices by pharmaceutical companies. Appropriate feedback from consumers helps to integrate social concerns into CSR strategies and to meet the expectations of key stakeholders, as opposed to simply pursuing CSR activities without understanding the needs of the society.

Therefore, we conducted this study 1) to investigate public awareness of CSR practices in the pharmaceutical industry in Korea, as an indicator of the public exposure CSR practices receive and the pharmaceutical industry’s success in attracting public attention to their CSR practices; 2) to examine the types of CSR activities preferred by the public; and 3) to understand the factors associated with those preferences. Based on these three objectives, we formulated the following study questions.

*Question 1*. *How interested are the respondents in CSR activities of pharmaceutical firms*?
*Question 2*. *Which CSR activities do the respondents prefer the most*?
*Question 3*. *Which CSR activities do the respondents expect to contribute the most to society*?
*Question 4*. *Do “healthy” and “patient” groups differ in their interests*, *preferences*, *and expectations regarding CSR activities of pharmaceutical firms*?
*Question 5*. *Do demographic factors explain the difference in respondents’ answers*?

We expect that this exploratory study will help the pharmaceutical industry to understand consumers’ responses to CSR activities, and identify the specific CSR activities that can positively impact consumers.

## Materials and methods

### Study subjects and data collection

In this study, online surveys were conducted on the general population and patient groups during March–April 2017. Representatives of the general population were recruited from panel members registered for a professional survey institution in Korea called “Embrain” (http://www.embrain.com/eng). This institution has a nationwide panel of more than one million people over the age of 13, which reflects the distributions of gender, age, and residence of the Korean population; therefore, it is representative of the general population [22]. A random sample comprising 1,000 panel members aged 20 years and over, who agreed to participate in the survey, was selected. Patient subjects were recruited from selected patient organizations that agreed to participate in this study, such as the Diabetic Patient Organization (http://www.dangnyo.or.kr/), the Liver Cancer Patient Organization (http://www.liverkorea.org/), and the Gastro-intestinal Stromal Cancer Patient Organization (http://cafe.daum.net/GIST). The target numbers of respondents for the general population and patients were 1,000 and 500, respectively; and 1,000 (100%) and 298 (59.6%) respondents completed the questionnaire, respectively.

### Measurements

In the self-administered questionnaire, we provided a short description of what CSR activities are, to help respondents have a consistent concept of CSR. We measured respondents’ attitudes toward CSR activities provided by pharmaceutical companies by asking about their interest in, awareness of, and personal experience with CSR activities provided by pharmaceutical companies. Respondents were also asked about whether they consider the pharmaceutical industry more or less active than other industries in implementing CSR activities. A total of 13 types of CSR activities were included in the survey, which were derived from the International Organization for Standardization’s definition of CSR [4], previous literature [16, 17], and the results of investigating CSR activities disclosed by the top 10 domestic and multinational pharmaceutical companies operating in Korea (S1 Table). Similar CSR activities were grouped together and conceptualized into the following six categories: “health promotion,” “improving work and welfare environment for employees,” “support for the underprivileged,” “social development,” “environmental protection,” and “emergency disaster relief support.” Categorization of these CSR activities was based on the categories utilized in the Social Contribution White Paper by the Federation of Korean Industries [23] and Droppert and Benette’s study [16]. For each type of CSR activity included in the survey, we asked respondents about their preferences for, and expectations of, its social contribution effects by utilizing a 10-point Likert-type scale, where higher scores indicated a greater preference or expected effects. In addition, demographic and health status information of all respondents was collected to examine their baseline characteristics. We developed a final survey questionnaire (S1 and S2 Files) after conducting a pilot test with adults of the general population and the representatives in the patient group.

### Ethics statement

All procedures involving human participants were performed in accordance with the ethical standards laid down by the Yonsei University Institutional Review Board (IRB) and the 1964 Helsinki declaration and its later amendments or comparable ethical standards. The survey methods were approved by the Yonsei University IRB (IRB No. 7001988-201703-HR-151-02). Informed consent was obtained from all individual participants, which included consent for publication.

### Data analysis

We analyzed the frequency distribution of each question representing respondents’ attitudes toward CSR activities in the pharmaceutical industry. For each type of CSR activity, the mean scores for respondents’ preferences for and expectations of social contribution effects were computed and compared between the general and patient groups.

To understand respondents’ characteristics associated with preferences for CSR by the pharmaceutical industry and to identify target groups whose needs were met by specific CSR activities, we conducted multivariate regression analyses with three empirical models. Model 1 was regressed on the mean preference score for all CSR activities included in the survey. We hypothesized that consumer preference and its associated characteristics would be different between CSR activities directly related to drug development and those that were unrelated. We therefore performed separate regression analyses using Model 2 and Model 3. Model 2 was regressed on the mean preference score for the CSR activities related to new drug development, such as “development of innovative drugs in untreated areas” and “support for research on new drug development,” which represent the core business and the fundamental contribution of the pharmaceutical industry that create shared value in our society. Finally, Model 3 was regressed on the mean preference score for the CSR activities not directly related to drugs, such as “community service activities” and “improving social issues.”

Our regression models were based on the consumer behavior model in marketing, which explains factors affecting consumer behavior. According to Kotler’s theory, major factors influencing consumer behavior are cultural, social, personal, and psychological [24]. Culture is defined as the characteristics of a group whose members share similar values, interests, and behaviors [25]. Social factors refer to a person’s family, work, residency, or reference groups [26]. It also represents a person’s social class involving income, education level, and living conditions. Personal factors are characteristics specific to a person, such as age, gender, and lifestyle [26]. Finally, psychological factors include motivation, perception, learning, beliefs, and attitudes.

Adopting these factors into our regression model to explain consumer preferences for CSR activities, we defined the covariates of our model as patient group, demographic characteristics, attitude toward CSR activities, and the expected effect of social contribution of CSR activities (Eq 1). The respondents’ inclusion in either the patient or the healthy group is accompanied by a corresponding cultural factor, since we believe that these two groups are heterogeneous in their values, interests, and behaviors regarding CSR. The demographic characteristics included gender, age, residence area, education level, marital status, and self-rated health status, which correspond to the social and personal factors of the consumer behavior model. Attitude and the expected effects of social contribution correspond to the psychological factors of the consumer behavior model. The specific variables reflecting consumer attitudes in our model included interest, awareness, and experience with CSR activities in the pharmaceutical industry, and their perception of pharmaceutical companies’ CSR activities in comparison to that of other industries.

$$Y=\beta_{0}+\beta_{1}(patient\;group)i+\beta_{2}(demographics)i+\beta_{3}(attitudes)i+\beta_{4}(expected\;effects)i+\varepsilon i$$ (Eq 1)

where

i = 1,…, n^th^ respondent

*Y*_1_ = mean preference score for all CSR activities (Model 1)

*Y*_2_ = mean preference score for CSR activities related to new drug development (Model 2)

*Y*_3_ = mean preference score for CSR activities not directly related to drugs (Model 3)

In addition, subgroup analysis was performed according to their experiences with CSR activities carried out by the pharmaceutical industry. All statistical analyses were performed using the SAS statistical program (release 9.4, SAS Institute Inc., Cary, NC, USA). The significance level was set at 5%.

## Results

### Demographic characteristics of the respondents

Among the 1,000 respondents from the general group, those who admitted to having one or more comorbidities such as cancer, chronic diseases, and rare diseases were reclassified as patients. Thus, the final study subjects consisted of 1,298 respondents: 546 healthy adults and 752 patients. The distribution of gender, residence area, and highest education level did not significantly differ between the two groups (Table 1). On the other hand, the percentage of patient respondents in their 50s or older was significantly higher than that of the healthy group by more than 10 percentage points (46.7% in the patient group vs. 37.7% in the healthy group). Approximately 87% of the patient group had chronic diseases, and about a quarter had cancer.

**Table 1:** Demographic and health characteristics of the survey respondents.

Characteristics	Number of respondents (%)			*p*-value^^^
	Overall	Healthy people	Patients	
Total	1,298 (100.0)	546 (100.0)	752 (100.0)	
Gender				0.104
Male	686 (52.9)	303 (55.5)	383 (50.9)	
Female	612 (47.1)	243 (44.5)	369 (49.1)	
Age (years)				< .001
20s	149 (11.5)	80 (14.7)	69 (9.2)	
30s	260 (20.0)	123 (22.5)	137 (18.2)	
40s	332 (25.6)	137 (25.1)	195 (25.9)	
50s	304 (23.4)	129 (23.6)	175 (23.3)	
60s or above	253 (19.5)	77 (14.1)	176 (23.4)	
Residency				0.331
Metropolitan	705 (54.3)	298 (54.6)	407 (54.1)	
City	292 (22.5)	131 (24.0)	161 (21.4)	
Rural	301 (23.2)	117 (21.4)	184 (24.5)	
Highest education				0.214
Middle school	24 (1.8)	10 (1.8)	14 (1.9)	
High school	271 (20.9)	119 (21.8)	152 (20.2)	
College graduate	820 (63.2)	353 (64.7)	467 (62.1)	
Graduate school or more	183 (14.1)	64 (11.7)	119 (15.8)	
Occupation				0.001**
Managers and professionals	324 (25.0)	119 (21.8)	205 (27.3)	
Clerks	400 (30.8)	196 (35.9)	204 (27.1)	
Service and sales workers	128 (9.9)	54 (9.9)	74 (9.8)	
Agriculture, fishery, and forestry workers	14 (1.1)	4 (0.7)	10 (1.3)	
Craft and related trades workers	30 (2.3)	10 (1.8)	20 (2.7)	
Elementary occupations	18 (1.4)	8 (1.5)	10 (1.3)	
Homemakers	208 (16.0)	81 (14.8)	127 (16.9)	
Students	58 (4.5)	35 (6.4)	23 (3.1)	
Unemployed	118 (9.1)	39 (7.1)	79 (10.5)	
Marital status				< .001^†^
Single	344 (26.5)	177 (32.4)	167 (22.2)	
Married	883 (68.0)	343 (62.8)	540 (71.8)	
Widowed	30 (2.3)	9 (1.6)	21 (2.8)	
Divorced	41 (3.2)	17 (3.1)	24 (3.2)	
Comorbidity*				
Cancer	-	-	186 (24.7)	
Rare disease	-	-	53 (7.0)	
Chronic disease	-	-	657 (87.4)	

* Duplicate responses were allowed.

^†^ p-value <0.01

^^^p-values were calculated for comparisons between the healthy and patient groups.

### Attitudes toward CSR

Overall, approximately 80% of the respondents expressed that they were somewhat or strongly interested in CSR activities in the pharmaceutical industry. Only 7.9% of the respondents reported to have experienced CSR activities in the pharmaceutical industry, and about three-quarters said they did not know or knew little about CSR activities in the pharmaceutical industry (Table 2). A statistically significantly higher proportion of the patient group (10.1%) had experience with CSR activities than the healthy group (4.8%, p<0.05). In addition, the proportion of respondents who thought that the pharmaceutical industry was more active in exercising CSR activities than other industries was significantly higher in the patient group (26.1%) than in the healthy group (17.0%, p<0.05).

**Table 2:** Attitudes toward corporate social responsibility activities of the pharmaceutical industry.

Types of attitude	Number of respondents (%)			*p*-value^^^
	Overall	Healthy people	Patients	
Total	1,298 (100.0)	546 (100.0)	752 (100.0)	
Degree of interest				0.027*
None	14 (1.1)	5 (0.9)	9 (1.2)	
Little	276 (21.3)	129 (23.6)	147 (19.5)	
Somewhat	871 (67.1)	369 (67.6)	502 (66.8)	
Strongly	137 (10.6)	43 (7.9)	94 (12.5)	
Degree of awareness				0.840
Not at all	107 (8.2)	48 (8.8)	59 (7.8)	
Little	842 (64.9)	354 (64.8)	488 (64.9)	
Somewhat	334 (25.7)	139 (25.5)	195 (25.9)	
Strongly	15 (1.2)	5 (0.9)	10 (1.3)	
Experience of CSR by pharmaceutical companies				< .001^†^
Yes	102 (7.9)	26 (4.8)	76 (10.1)	
No	1,196 (92.1)	520 (95.2)	676 (89.9)	
Comparison with other industries				0.002^†^
Pharma industry is less active	97 (7.5)	41 (7.5)	56 (7.4)	
Pharma industry is similar	557 (42.9)	251 (46.0)	306 (40.7)	
Pharma industry is more active	289 (22.3)	93 (17.0)	196 (26.1)	
No idea	355 (27.3)	161 (29.5)	194 (25.8)	

CSR denotes corporate social responsibility.

* p-value <0.05

^†^ p-value <0.01

^^^p-values were calculated for comparisons between the healthy and patient groups.

### Preferences for types of CSR activities

Respondents were found to have positive preferences, as shown by mean scores higher than five, for all types of CSR activities included in the survey (Table 3). Among the six categories, the most preferred CSR activities were identified as those related to “promoting public health” (mean score 6.36) and “emergency disaster relief support” (6.34). However, respondents had relatively low preferences for activities not directly related to drugs, such as “improving work and welfare environment for employees” (5.89), “support for the underprivileged” (5.93), “social development” (5.90), and “environmental protection” (5.81). Relative preferences expressed in rankings of CSR activities between the healthy and the patient groups were similar; however, there was a difference in the absolute preferences as expressed by the mean score. In the healthy group, the difference between the highest and lowest mean score among individual CSR activities was 0.71 (6.63 vs. 5.92), whereas in the patient group, the difference was 1.24 (6.84 vs. 5.60). Among the 13 types of individual CSR activities, there were only four activities wherein the groups showed significant differences. The patient group expressed significantly higher preferences for only one activity, which was “support for research on new drug development” (6.84 in the patient group vs. 6.59 in the healthy group, p<0.05). Meanwhile, the healthy group showed significantly higher preferences for the remaining three activities, none of which are directly related to drugs: “community service activities” (6.14 by the healthy group vs. 5.83 by the patient group, p<0.05), “improving social issues” (5.92 vs. 5.60, p<0.05), and “environmental protection” (5.95 vs. 5.71, p<0.05).

**Table 3:** Preferences for types of CSR activities in the pharmaceutical industry.

Types of CSR activities		Mean score (standard deviation)^¶^			*p*-value^^^	Ranking	
		Overall (n = 1,298)	Healthy people (n = 546)	Patients (n = 752)		Healthy people (n = 546)	Patients (n = 752)
**Total**		**6.06 (1.79)**	**6.11 (1.69)**	**5.98 (1.86)**	**0.310**	-	-
**Promoting public health**		**6.36 (1.82)**	**6.34 (1.75)**	**6.37 (1.87)**	**0.716**	**[1]**	**[1]**
	Development of innovative drugs in untreated areas	6.74 (2.00)	6.63 (1.89)	6.82 (2.08)	0.094	1	2
	Support for research on new drug development	6.74 (1.98)	6.59 (1.91)	6.84 (2.02)	0.023^*^	2	1
	Offering free or low-priced drugs for vulnerable patients	6.11 (2.39)	6.17 (2.24)	6.06 (2.49)	0.431	6	7
	Support activities to improve treatment effectiveness of drug therapy (e.g., open lecture for patients, exercise program for diabetic patients, etc.)	6.25 (2.13)	6.27 (2.03)	6.23 (2.21)	0.726	4	4
	Improving disease awareness (e.g., AIDS and mental health campaigns, smoking cessation education, etc.)	6.20 (2.10)	6.23 (1.97)	6.18 (2.18)	0.667	5	5
	Providing up-to-date medical and drug information	6.11 (1.99)	6.12 (1.84)	6.10 (2.10)	0.867	9	6
**Improving work and welfare environment for employees**		**5.89 (1.87)**	**5.95 (1.73)**	**5.84 (1.96)**	**0.287**	**[5]** 11	**[3]** 9
**Support for the underprivileged**		**5.93 (2.05)**	**6.08 (1.92)**	**5.83 (2.13)**	0.029^*^	**[3]**	**[4]**
	Community service activities not directly related to drugs (e.g., support for the elderly who live alone, delivery of free briquettes, etc.)	5.96 (2.14)	6.14 (2.01)	5.83 (2.22)	0.009^†^	8	10
	Operation of educational programs and scholarship support	5.90 (2.10)	6.01 (1.99)	5.82 (2.16)	0.103	10	11
**Social development**		**5.90 (2.01)**	**6.03 (1.91)**	**5.80 (2.08)**	0.042^*^	**[4]**	**[5]**
	Increasing number of jobs by promoting employment in the pharmaceutical industry	6.06 (2.11)	6.15 (1.97)	6.00 (2.20)	0.211	7	8
	Improving social issues not directly related to drugs	5.73 (2.10)	5.92 (1.96)	5.60 (2.20)	0.008^†^	13	13
**Environmental protection** (e.g., energy saving projects)		**5.81 (2.15)**	**5.95 (2.01)**	**5.71 (2.24)**	0.042^*^	**[5]** 11	**[6]** 12
**Emergency disaster relief support**		**6.34 (2.23)**	**6.31 (2.10)**	**6.35 (2.33)**	**0.747**	**‘[2]** 3	**[2]** 3

^*^ **p-value <0.05**

^**†**^ **p-value <0.01**

^¶^ Preference was measured on a 10-point Likert-type scale, where a higher score indicates a higher preference.

Numbers in parentheses represent ranking by preference score across six categories of CSR activities.

^^^p-values were calculated for comparisons between the healthy and patient groups.

### Expectations regarding social contribution effects of CSR activities

**Fig 1:**
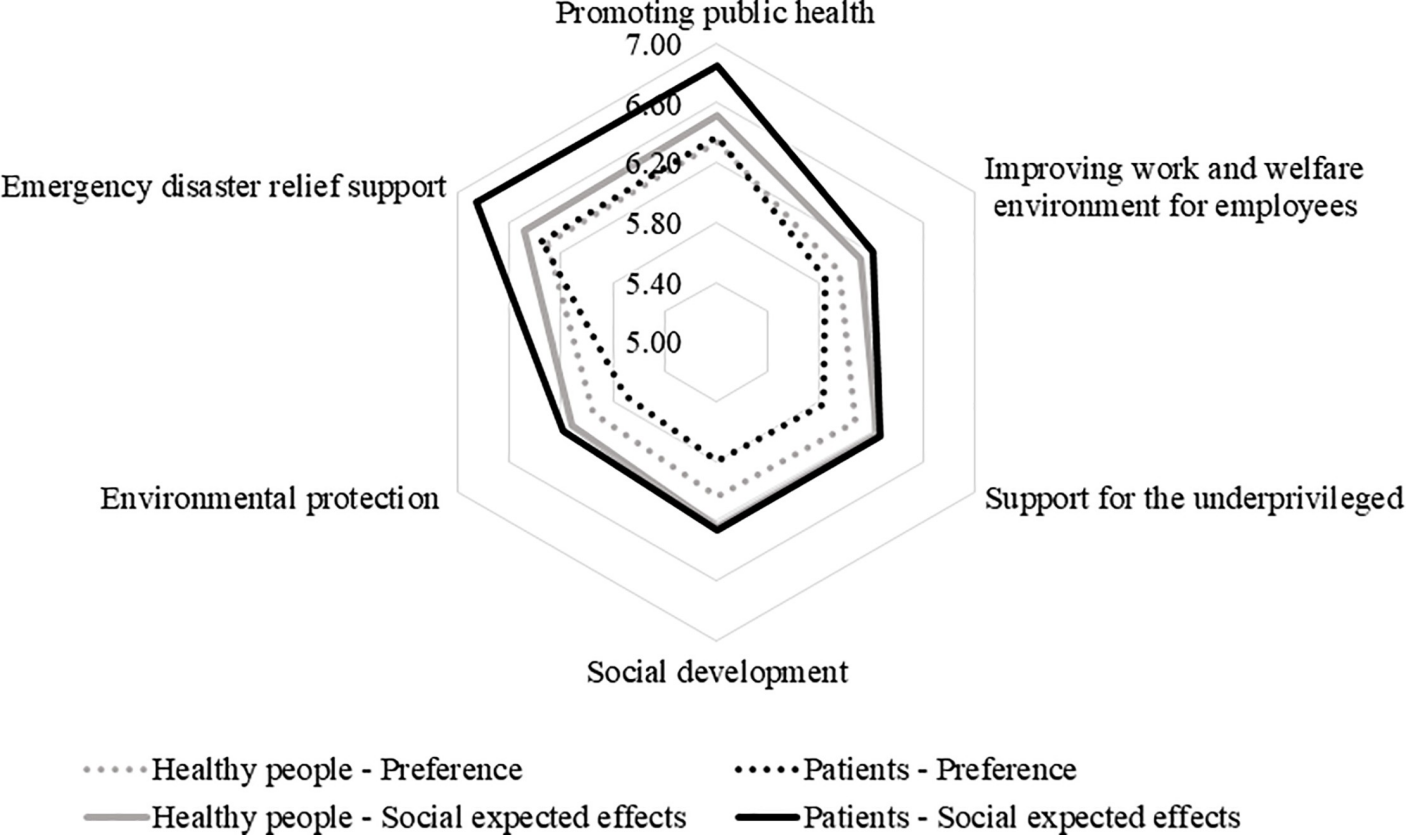
Mean scores of preferences and expectations regarding the social contribution effects of corporate social responsibility (CSR) activities in the pharmaceutical industry.

Overall, respondent answers indicated that the mean score of expectations regarding social contribution effects of CSR activities was six or higher for all CSR items (Table 4) and that it was higher than the mean score of the preferences (Fig 1). Among the six categories, the highest mean expectation score was observed for “promoting health” (6.71) and “emergency disaster relief support” (6.70). Its tendency was the same as that of the preference score, and the rankings between the two groups were similar.

**Table 4:** Expectations regarding social contribution effects of CSR activities of pharmaceutical companies.

Description of CSR		Mean score (standard deviation) ^¶^			*p*-value^^^	Ranking	
		Overall (n = 1,298)	Healthy people (n = 546)	Patients (n = 752)		Healthy people (n = 546)	Patients (n = 752)
**Total**		**6.37 (1.82)**	**6.29 (1.77)**	**6.44 (1.85)**	**0.145**	-	-
**Promoting public health**		**6.71 (1.87)**	**6.52 (1.83)**	**6.85 (1.89)**	0.002*	**[1]**	**[2]**
	Development of innovative drugs in untreated areas	7.02 (2.04)	6.73 (1.96)	7.24 (2.07)	< .001^†^	1	1
	Support for research on new drug development	6.97 (2.03)	6.72 (1.99)	7.16 (2.04)	< .001^†^	2	2
	Offering free or low-priced drugs for vulnerable patients	6.68 (2.23)	6.49 (2.12)	6.82 (2.30)	0.010^†^	3	4
	Support activities to promote treatment effectiveness beyond drugs (e.g., open lecture for patients, exercise program for diabetic patients, etc.)	6.63 (2.07)	6.48 (2.03)	6.74 (2.09)	0.027*	5	5
	Improving disease awareness activities (e.g., AIDS and mental health campaigns, smoking cessation education, etc.)	6.48 (2.04)	6.37 (1.99)	6.56 (2.07)	0.104	7	7
	Providing up-to-date medical and drug information	6.46 (2.02)	6.30 (1.91)	6.58 (2.09)	0.106*	8	6
**Improving work and welfare environment for employees**		**6.17 (1.95)**	**6.11 (1.86)**	**6.21 (2.02)**	**0.368**	**[6] 11**	**[5] 11**
**Support for the underprivileged**		**6.25 (2.02)**	**6.24 (1.93)**	**6.26 (2.09)**	**0.911**	**[3]**	**[3]**
	Community service activities not directly related to drugs (e.g., support for the elderly who live alone, delivery of briquettes, etc.)	6.28 (2.12)	6.29 (2.03)	6.26 (2.18)	0.835	9	9
	Operation of educational programs and scholarship support	6.22 (2.05)	6.20 (1.95)	6.25 (2.12)	0.664	10	10
**Social development**		**6.25 (1.97)**	**6.24 (1.88)**	**6.26 (2.03)**	**0.802**	**[3]**	**[3]**
	Increasing number of jobs by promoting employment from pharmaceutical industry	6.39 (2.07)	6.38 (1.93)	6.40 (2.16)	0.862	6	8
	Improving social issues not directly related to drugs	6.11 (2.04)	6.09 (1.97)	6.13 (2.09)	0.759	13	13
**Environmental protection (e.g., energy saving project)**		**6.15 (2.08)**	**6.12 (2.01)**	**6.18 (2.13)**	**0.616**	**[5] 12**	**[6] 12**
**Emergency disaster relief support**		**6.70 (2.16)**	**6.49 (2.09)**	**6.86 (2.19)**	**0.003**^**†**^	**[2] 3**	**[1] 3**

^*^ **p-value <0.05**

^†^ **p-value <0.01**

^¶^ Expectation was measured on a 10-point Likert-type scale, where a higher score indicates a higher expectation.

Numbers in parentheses represent ranking by expectation score across six categories of CSR activities.

^^^p-values were calculated for comparisons between the healthy and patient groups.

Among the 13 individual CSR items, the highest mean scores were assigned to items directly related to drug development in both the healthy and the patient groups. Especially in the patient group, the mean expected effects score for “development of innovative drugs in untreated areas” was higher than 7, implying that patients have firm beliefs about the contribution of innovative drugs to our society. As in the preference score, the difference between the highest and lowest mean expected effects score was larger in the patient group than in the healthy group. The difference in the healthy group was 0.64 (6.73 vs. 6.09), whereas it was 1.11 (7.24 vs. 6.13) in the patient group.

### Factors explaining preferences for CSR activities

The regression analysis results showed that the patient group had higher preferences for CSR activities related to new drug development (β = 0.20 from Model 2) and lower preferences for CSR activities not directly related to drugs (β = -0.36 from Model 3) than did the healthy group. Regardless of the type of CSR activity, women showed lower preferences (β = -0.19 ~ -0.33). Having experience with CSR activities significantly increased preferences for all types of CSR activities (β = 0.26 from Model 1). If the respondents thought that the pharmaceutical industry was more active than other industries in exercising CSR activities, the preference scores were higher in all models. The higher the score for expected effects of social contributions, the more positive the preference score. As the score of expected effects increased by one point, the preference score increased by 0.73, 0.54, and 0.79 points in Models 1, 2, and 3, respectively (Table 5).

**Table 5:** Regression analysis results of factors influencing respondent preferences for CSR activities.

	β coefficients (SE)		
	Model 1. Preference score of total CSR items	Model 2. Preference score of CSR items related to new drug development	Model 3 Preference score of CSR items not directly related to drugs
***Group***			
Healthy [reference]	-	-	-
Patients	-0.18 (0.07)^†^	0.20 (0.09)^*^	-0.36 (0.08)^†^
***Demographics***			
Gender			
Male [reference]	-	-	-
Female	-0.19 (0.07)^†^	-0.33 (0.09)^†^	-0.20 (0.08)^*^
Age group			
20s–40s [reference]	-	-	-
50s or above	-0.02 (0.07)	0.17 (0.10)	-0.01 (0.09)
Residency			
Metropolitan [reference]	-	-	-
City	0.03 (0.08)	0.01 (0.11)	0.11 (0.10)
Rural	-0.01 (0.08)	-0.10 (0.11)	0.01 (0.10)
Highest education			
High school or below [reference]	-	-	-
More than college graduates	0.01 (0.08)	0.10 (0.11)	0.04 (0.10)
Marital status			
Live alone [reference]	-	-	-
Live with spouse	-0.12 (0.07)	0.01 (0.10)	-0.10 (0.09)
Self-rated health status			
Poor [reference]	-	-	-
Fair	0.17 (0.11)	0.13 (0.15)	0.04 (0.13)
Good or very good	0.11 (0.11)	0.31 (0.15)^*^	-0.11 (0.10)
***Attitudes***			
Interest			
No [reference]	-	-	-
Yes	0.01 (0.08)	0.30 (0.01)	-0.11 (0.10)
Awareness			
No [reference]	-	-	-
Yes	0.04 (0.08)	0.01 (0.11)	0.06 (0.10)
Experience			
No [reference]	-	-	-
Yes	0.26 (0.13)^*^	-0.07 (0.17)	0.22 (0.16)
Comparison with other industries			
Pharma industry is less active [reference]	-	-	-
Pharma industry is similar	0.39 (0.09)^†^	0.45 (0.12)^†^	0.41 (0.11)^†^
Pharma industry is more active	0.56 (0.14)^†^	0.85 (0.19)^†^	0.51 (0.17)^†^
No idea	0.28 (0.10) ^*^	0.17 (0.13)	0.35 (0.12)^†^
***Expected effect***			
Total scores	0.73 (0.02)^†^	0.54 (0.03)^†^	0.79 (0.02)^†^
Adjusted R-squared	0.576	0.317	0.515

SE denotes standard errors.

* p-value <0.05

^†^ p-value <0.01

As shown in Table 6, there were 26 respondents (4.8%) in the healthy group and 76 respondents (10.1%) in the patient group who had experienced CSR activities by the pharmaceutical industry. In the healthy group, there were no differences in the preferences and expected effects score according to CSR experience. Interestingly, in the patient group, those with experience of CSR showed significantly higher preference scores for all six categories of CSR activities. However, no difference within the patient group was observed in the expected effects scores of those with CSR experience versus those without.

**Table 6:** Preferences for and expectations of social contribution effects of CSR according to respondents’ experiences of CSR activities of pharmaceutical companies.

Description of social contribution	Healthy people (n = 546)			Patients (n = 752)		
	Mean score (Std)		*p*-value^	Mean score (Std)		*p*-value^
Experience with CSR by pharma	Yes (n = 26)	No (n = 520)		Yes (n = 76)	No (n = 676)	
**Preference**						
Total	6.21 (1.66)	6.11 (1.69)	0.751	6.57 (1.74)	5.92 (1.86)	0.004^†^
Promoting public health	6.51 (1.73)	6.33 (1.76)	0.598	6.94 (1.75)	6.31 (1.88)	0.005^†^
Improving work and welfare environment for internal employees	6.27 (1.64)	5.94 (1.74)	0.340	6.37 (1.61)	5.78 (1.99)	0.013*
Support for the underprivileged	6.27 (1.83)	6.07 (1.93)	0.603	6.33 (2.21)	5.77 (2.11)	0.030*
Social development	6.00 (1.76)	6.03 (1.91)	0.930	6.43 (1.92)	5.73 (2.08)	0.005^†^
Environmental protection (e.g., energy saving projects)	6.00 (1.88)	5.95 (2.02)	0.905	6.36 (2.18)	5.64 (2.24)	0.008^†^
Emergency Disaster Relief Support	6.23 (2.05)	6.32 (2.10)	0.838	6.97 (2.14)	6.28 (2.34)	0.014*
**Expected effects**						
**Total**	6.26 (1.57)	6.29 (1.78)	0.931	6.70 (1.93)	6.41 (1.83)	0.191
Promoting public health	6.31 (1.59)	6.53 (1.84)	0.565	7.20 (1.96)	6.81 (1.87)	0.086
Improving work and welfare environment for internal employees	6.04 (1.93)	6.11 (1.86)	0.842	6.61 (2.03)	6.16 (2.01)	0.071
Support for the underprivileged	6.29 (1.63)	6.24 (1.94)	0.902	6.37 (2.18)	6.24 (2.08)	0.619
Social development	6.33 (1.70)	6.23 (1.89)	0.801	6.50 (2.12)	6.24 (2.02)	0.285
Environmental protection (e.g., energy saving project)	6.35 (1.70)	6.11 (2.02)	0.559	6.39 (2.26)	6.16 (2.12)	0.354
Emergency Disaster Relief Support	6.23 (1.75)	6.51 (2.10)	0.510	7.12 (2.13)	6.83 (2.20)	0.275

* p-value <0.05

^†^ p-value <0.01

Std denotes standard deviations

^P-values were calculated for comparisons between those with and those without experience.

## Discussion

To the best of our knowledge, this is the first empirical study to assess public perceptions of and attitudes toward CSR activities implemented in the pharmaceutical industry and to identify specific CSR activities favored by the public. Our results show that respondents had interest in and positive attitudes toward CSR activities of pharmaceutical companies; however, they had little experience with and awareness of those practices. Future studies should identify the underlying reasons such as whether this was because the CSR activities were too infrequent or because the companies did not effectively promote their activities. Both the healthy and the patient groups had similar priorities in their preferences for and expectations of social contribution effects of the preferred CSR items. They had a high preference for CSR items of “development of innovative drugs in untreated areas” and “support for research on new drug development,” and they showed a relatively low preference for items of “environmental protection” and “employee welfare.” This study discovered that activities related to “the improvement of human health,” the ultimate purpose of the pharmaceutical industry, were the main method through which respondents wanted the pharmaceutical industry to contribute to society. This is in contrast to the results in the food industry and the banking industry, where environmental responsibility and customer-centered CSR activities relating to staff’s attitudes or feedbacks, respectively, are highly preferred [11, 27]. These results indicate that consumer demand and preferences for CSR activities differ by industry. Therefore, it is crucial to select CSR items that meet public expectations and are relevant to the industry, because unsuitable CSR activities can lead to poor performance [28]. In this regard, the results of this study suggest that the public expects the pharmaceutical industry to promote health through new drug development, which is drug companies’ core business. This result implies that the public wants pharmaceutical companies to invest in new drug development in currently untreated areas, such as in rare diseases, as a form of philanthropy-type CSR activity that returns benefits to society.

Interestingly, the respondents showed higher scores for expectations of social contribution effects than for preferences for any individual CSR item. We thus learned that although Koreans’ personal preferences for CSR activities by drug companies were not very keen, their belief that those activities can help improve the condition of our society was very strong.

It should be noted that there were prominent discrepancies between the healthy and the patient groups in many aspects regarding the drug companies’ CSR activities. The patient group was more definite than the healthy group in their scoring of preferences for CSR items, and they responded with higher preference scores for CSR items related to new drug development. This finding supports that of Šramová and Kučeráková [29] and can be explained as follows. First, compared to the healthy group, the patient group had more experiences with the CSR activities implemented by pharmaceutical companies and more strongly agreed that pharmaceutical companies are more active in implementing CSR activities than other industries (Table 2). Based on these responses, we assumed that pharmaceutical companies’ CSR activities in Korea targeted patients more than the general population. Second, the differences in scores for both preferences and expected effects across CSR activities was higher within the patient group than within the healthy group (Tables 3 and 4), implying that the patient group had more explicit priorities across various CSR activities. For the patient group, the preferences for and expected effects of CSR activities related to drug development and health promotion were substantially higher than those for the unrelated activities. Third, while having experience with CSR activities does not seem to affect preferences for CSR activities among healthy people, it significantly increases preferences among patients.

The results of the regression analysis of the factors affecting the degree of preference for CSR activities based on the consumer behavior model indicate that patient group (a cultural factor), gender (a personal factor), and attitudes toward and expected effects of CSR (a psychological factor) were significant factors in all of the three models (Model 1, 2, and 3). It was interesting that respondents’ demographic characteristics were not significant factors, except for gender. This result is similar to that obtained in McWilliams’s study [14]. Women had a significantly lower preference for CSR activities than men, regardless of the type of CSR activity. Although further research on why women have a lower preference for CSR activities is needed, it is noteworthy that gender differences revealed in the CSR activities implemented by the pharmaceutical industry is likewise echoed in other social preferences [30].

According to a recent Gallup Poll, the reputation of the pharmaceutical industry in America has been getting worse [31]. Only 28% of the respondents had a positive perception, while 51% had a negative view of the pharmaceutical industry. This result is the worst during the 16 years that it has been tracked. Silverman analyzed this phenomenon as follows. The public has steadily unmet needs for hard-to-treat diseases; however, it is also uncomfortable with business ethics issues, such as the regulation of drug prices and the management of drug safety [32]. Implementing appropriate and customized CSR activities can improve the reputation of pharmaceutical companies. The two findings of this study have significant implications for the pharmaceutical industry: “development of innovative drugs in untreated areas” and “support for research on new drug development” are the CSR activities people prefer regardless of their health status; and CSR experience is a key factor influencing preference of CSR activity. The pharmaceutical industry should carry out and actively promote the kinds of CSR activities that their customers prefer.

The results of our study should be interpreted with caution. Since approximately 85% of the respondents from the patient group had chronic diseases such as hypertension and diabetes mellitus, the results of the patient group in this study are limited to representing the perceptions of patients with chronic conditions. Patients with different conditions, such as cancer, may have different perceptions of the drug industry’s CSR activities. In addition, since the patient group is older than the healthy group, thy might have had more opportunities to experience CSR due to their relatively longer life experience. If the relatively older patient group had a positive outcome from pharmaceutical companies’ CSR activities, this could result in an increased awareness/perception of pharmaceutical companies in the patient group and an increase in the magnitude of the social expectation effect for this CSR item. Finally, the respondents’ positive preferences for CSR activities may not necessarily have a positive impact on consumers.

## Conclusions

Although company websites and annual reports indicate that pharmaceutical companies in Korea undertake a variety of CSR activities, our survey results reveal that the Korean public do not have much experience with or awareness of such CSR activities. Focusing on CSR activities that successfully accommodate public preferences, such as health-promotion related activities, might be an effective strategy to improve public awareness of CSR activities provided by pharmaceutical companies. A key finding is that both the healthy and the patient groups’ number one preference for pharmaceutical companies’ CSR activities was related to new drug development, such as the “development of innovative drugs in untreated areas” and “support for research on new drug development.” This finding reveals that our society thinks that CSR activities provided by pharmaceutical companies should first and foremost pursue the pharmaceutical industry’s intrinsic mission: to fulfill unmet medical needs by developing new drugs. Unlike healthy people, patients were influenced by their experiences with CSR activities, and they showed significant associations between their CSR experiences and preferences for CSR activities.

In sum, the present study enriches the literature on the factors that explain consumer preferences for CSR activities. In addition, by identifying the CSR activities preferred by the public, our study helps pharmaceutical companies behave more strategically in developing CSR activities that have the potential to benefit the public. We expect that our findings will also help policy makers design the necessary policies to support CSR activities in the pharmaceutical industry, which will improve our society.

## Supporting information

S1 Table Top 10 domestic and multinational pharmaceutical companies investigated. (DOCX)

S1 File Survey questionnaire (English). (DOCX)

S2 File Survey questionnaire (Korean). (DOCX)
