# Supplementary material for: Public preferences for corporate social responsibility activities in the pharmaceutical industry: Empirical evidence from Korea
Source: PLoS One. 2019 Aug 20;14(8):e0221321. doi: 10.1371/journal.pone.0221321 (PMC6701779; doi:10.1371/journal.pone.0221321)
Supplement: S1 Table — (DOCX) [file pone.0221321.s001.docx]

**S1 Table. Top 10 domestic and multinational pharmaceutical companies investigated**

| No | Company Name | Website |
| --- | --- | --- |
| *Domestic companies* | | |
| 1 | Yuhan Corporation | https://www.yuhan.co.kr/ |
| 2 | Hanmi Pharm | http://www.hanmiscience.co.kr/ |
| 3 | Green Cross | http://www.greencross.co.kr |
| 4 | Daewoong Pharm | www.daewoong.co.kr |
| 5 | Jeil Pharm | www.jeilpharm.co.kr |
| 6 | Chong Kun Dang | http://www.ckdpharm.com |
| 7 | Kwangdong Pharm | http://www.ekdp.com |
| 8 | Dong-A ST | http://www.donga-st.com |
| 9 | Celltrion | http://www.celltrionsw.com/ |
| 10 | CJ Healthcare | www.cjp.co.kr |
| *Multinational companies* | | |
| 1 | Novartis Korea | https://www.novartis.co.kr/ |
| 2 | Pfizer Korea | http://www.pfizer.co.kr/ |
| 3 | Sanofi Korea | http://www.sanofi.co.kr/ |
| 4 | MSD Korea | http://www.msd-korea.com/ |
| 5 | Roche Korea | http://www.roche.co.kr |
| 6 | GSK Korea | http://kr.gsk.com/ |
| 7 | Gilead Korea | http://www.gilead.com/ |
| 8 | Johnson & Johnson (Janssen Korea) | http://www.janssen.com/korea/ |
| 9 | Astrazeneca Korea | http://www.astrazeneca.co.kr/ |
| 10 | AbbVie Korea | http://www.abbvie.co.kr |
