# Supplementary material for: Public preferences for corporate social responsibility activities in the pharmaceutical industry: Empirical evidence from Korea
Source: PLoS One. 2019 Aug 20;14(8):e0221321. doi: 10.1371/journal.pone.0221321 (PMC6701779; doi:10.1371/journal.pone.0221321)
Supplement: S1 File — (DOCX) [file pone.0221321.s002.docx]

**S1 File. Survey questionnaire (English)**

Please answer the following questions after reading the description of “corporate social responsibility” activities in the below box.

| Corporate social responsibility (CSR) refers to business operations involving initiatives that benefit society.  CSR encompasses activities that integrate social and environmental concerns in their business operations on a voluntary basis and creates shared values for all stakeholders including consumers, communities, governments, and corporations with a sustainable development.  Specific examples of a business's CSR activities include the following: complying with the law and ethical standards in business practice, developing innovative products that meet social needs and improving the quality of life of the public (innovation and growth), donating to national and local charities (philanthropy), doing good deeds without expecting anything in return (volunteering), treating employees fairly and ethically (ethical labor practices), and implementing greener business operations (environmental efforts) |
| --- |

|  | None | Little | Somewhat | Strongly |
| --- | --- | --- | --- | --- |
| 1. To what extent are you interested in CSR activities in general? |  |  |  |  |
| 2. To what extent are you aware of pharmaceutical company’s CSR activities? |  |  |  |  |

3. Have you experienced pharmaceutical company’s CSR activities?

① Yes ② No

4. Compared to other industries, how actively do you think the pharmaceutical industry implements CSR activities?

① More active than other industries

② As active as other industries

③ Less active than other industries

④ Don’t know

5. The following are examples of pharmaceutical company’s CSR activities. Please grade each activity from 1 to 10 based on your preference. 1 means very strongly negative and 10 means very strongly positive.

| Types of CSR activities | 1 | 2 | 3 | 4 | 5 | 6 | 7 | 8 | 9 | 10 |
| --- | --- | --- | --- | --- | --- | --- | --- | --- | --- | --- |
| **Promoting public health** | | | | | | | | | | |
| Development of innovative drugs in untreated areas |  |  |  |  |  |  |  |  |  |  |
| Support for research on new drug development |  |  |  |  |  |  |  |  |  |  |
| Offering free or low-priced drugs for vulnerable patients |  |  |  |  |  |  |  |  |  |  |
| Support activities to improve treatment effectiveness of drug therapy (e.g., open lecture for patients, exercise program for diabetic patients, etc.) |  |  |  |  |  |  |  |  |  |  |
| Improving disease awareness (e.g., AIDS and mental health campaigns, smoking cessation education, etc.) |  |  |  |  |  |  |  |  |  |  |
| Providing up-to-date medical and drug information |  |  |  |  |  |  |  |  |  |  |
| **Improving work and welfare environments for employees** |  |  |  |  |  |  |  |  |  |  |
| **Support for the underprivileged** | | | | | | | | | | |
| Community service activities not directly related to drugs (e.g., support for elderly living alone, delivery of free briquettes, etc.) |  |  |  |  |  |  |  |  |  |  |
| Operation of educational programs and scholarship support |  |  |  |  |  |  |  |  |  |  |
| **Social development** |  |  |  |  |  |  |  |  |  |  |
| Increasing number of jobs by promoting employment from pharmaceutical industry |  |  |  |  |  |  |  |  |  |  |
| Improving social issues not directly related to drugs |  |  |  |  |  |  |  |  |  |  |
| **Environmental protection** (e.g., energy saving projects) |  |  |  |  |  |  |  |  |  |  |
| **Emergency disaster relief support** |  |  |  |  |  |  |  |  |  |  |

6. Please grade each CSR activity from 1 to 10 based on your expectation for the social contribution. 1 means very strongly negative and 10 means very strongly positive.

| Types of CSR activities | 1 | 2 | 3 | 4 | 5 | 6 | 7 | 8 | 9 | 10 |
| --- | --- | --- | --- | --- | --- | --- | --- | --- | --- | --- |
| **Promoting public health** | | | | | | | | | | |
| Development of innovative drugs in untreated areas |  |  |  |  |  |  |  |  |  |  |
| Support for research on new drug development |  |  |  |  |  |  |  |  |  |  |
| Offering free or low-priced drugs for vulnerable patients |  |  |  |  |  |  |  |  |  |  |
| Support activities to improve treatment effectiveness of drug therapy (e.g., open lecture for patients, exercise program for diabetic patients, etc.) |  |  |  |  |  |  |  |  |  |  |
| Improving disease awareness (e.g., AIDS and mental health campaigns, smoking cessation education, etc.) |  |  |  |  |  |  |  |  |  |  |
| Providing up-to-date medical and drug information |  |  |  |  |  |  |  |  |  |  |
| **Improving work and welfare environments for employees** |  |  |  |  |  |  |  |  |  |  |
| **Support for the underprivileged** | | | | | | | | | | |
| Community service activities not directly related to drugs (e.g., support for elderly living alone, delivery of free briquettes, etc.) |  |  |  |  |  |  |  |  |  |  |
| Operation of educational programs and scholarship support |  |  |  |  |  |  |  |  |  |  |
| **Social development** |  |  |  |  |  |  |  |  |  |  |
| Increasing number of jobs by promoting employment from pharmaceutical industry |  |  |  |  |  |  |  |  |  |  |
| Improving social issues not directly related to drugs |  |  |  |  |  |  |  |  |  |  |
| **Environmental protection** (e.g., energy saving projects) |  |  |  |  |  |  |  |  |  |  |
| **Emergency disaster relief support** |  |  |  |  |  |  |  |  |  |  |
