# Supplementary material for: Public preferences for corporate social responsibility activities in the pharmaceutical industry: Empirical evidence from Korea
Source: PLoS One. 2019 Aug 20;14(8):e0221321. doi: 10.1371/journal.pone.0221321 (PMC6701779; doi:10.1371/journal.pone.0221321)
Supplement: S2 File — (DOCX) [file pone.0221321.s003.docx]

**S2 File. Survey questionnaire (Korean)**

다음에서 설명하는 기업의 ‘**사회기여 및 공헌활동**’의 정의를 잘 읽고 문항에 응답해 주십시오.

기업의 사회기여 및 공헌활동이란 기업이 사회의 기대와 요구에 충족하기 위해 사회의 공익에 대한 책임을 실천하는 모든 활동을 의미합니다.

가령, 기업이 합법적·윤리적으로 기업을 운영하는 것, 혁신적 제품의 지속적 개발로 사회구성원의 삶의 질 개선에 기여하는 것과 같은 기업 본연의 활동을 비롯하여,

직원들의 업무 및 복지환경 개선, 지역사회 소외계층 지원, 교육 및 문화예술분야 후원, 환경 보호, 응급 재난구호 지원 활동 등이 포함되며,

이러한 활동을 통해 소비자, 지역사회, 정부, 기업이 모두 발전하는 공유 가치를 창출하는 것을 의미합니다.

1. 귀하는 기업의 사회기여 및 공헌활동에 대해 평소 어느 정도 관심을 가지고 계십니까?

① 전혀 관심 없다.

② 관심이 없는 편이다.

③ 관심이 있는 편이다.

④ 매우 관심 있다.

2. 귀하는 **제약회사**가 하고 있는 사회기여 및 공헌활동에 대해 평소 어느 정도 알고 계십니까?

① 전혀 모르고 있다.

② 모르는 편이다.

③ 알고 있는 편이다.

④ 매우 잘 알고 있다.

3. 귀하는 **제약회사**가 제공하는 사회기여 및 공헌활동의 대상이 되거나 경험하신 적이 있습니까?

① 있다.

② 없다.

4. 귀하는 **제약회사**가 다른 산업과 비교하여 얼마나 활발하게 사회기여 및 공헌활동을 하고 있다고 생각하십니까?

① 다른 산업에 비해 **더** 활발하게 하고 있다.

② 다른 산업과 비슷하게 하고 있다.

③ 다른 산업에 비해 **덜** 활발하게 하고 있다.

④ 잘 모르겠다.

5. 다음은 **제약회사**의 사회기여 및 공헌활동 사례입니다. 각 활동별로 귀하의 **선호도**를 1~10점에서 선택해 주십시오. (10점으로 갈수록 선호도가 강함)

| 사회기여 및 공헌활동 | 1 | 2 | 3 | 4 | 5 | 6 | 7 | 8 | 9 | 10 |
| --- | --- | --- | --- | --- | --- | --- | --- | --- | --- | --- |
| **국민건강 증진** | | | | | | | | | | |
| 미치료 영역에서의 혁신적인 의약품 개발 |  |  |  |  |  |  |  |  |  |  |
| 신약개발 연구 지원 (예: 연구개발비 투자, 산학 공동연구 지원 등) |  |  |  |  |  |  |  |  |  |  |
| 취약계층 환자를 위해 자사 제품을 무료 혹은 저가로 제공 |  |  |  |  |  |  |  |  |  |  |
| 환자의 치료효과 향상을 위해 의약품 외 지원 활동(예: 환자대상 공개강좌, 당뇨환자에게 운동프로그램 지원 등) |  |  |  |  |  |  |  |  |  |  |
| 일반인 대상 질병 인식 개선활동 (예: 에이즈 캠페인, 정신건강 바로 알기 캠페인, 금연 교육 등) |  |  |  |  |  |  |  |  |  |  |
| 최신 의학 정보 제공 |  |  |  |  |  |  |  |  |  |  |
| **내부직원의 업무 및 복지환경 개선** | | | | | | | | | | |
| 제약회사 직원(내부고객)의 업무 및 복지환경 증진을 통한 삶의 질 개선 |  |  |  |  |  |  |  |  |  |  |
| **소외계층 지원** | | | | | | | | | | |
| 제약회사 제품과 직접 관련 없는 지역사회 봉사활동 (예: 연탄배달, 독거노인 돌봄 지원, 등) |  |  |  |  |  |  |  |  |  |  |
| 교육 프로그램 운영 (예: 다문화가정 학습 지원, 심리치유 프로그램), 장학금 지원 |  |  |  |  |  |  |  |  |  |  |
| **사회발전** |  |  |  |  |  |  |  |  |  |  |
| 제약회사의 고용 증진을 통한 일자리 증가 |  |  |  |  |  |  |  |  |  |  |
| 제약회사 제품과 직접 관련 없는 사회적 이슈 개선활동 (예: 장애인 인식 개선, 문화재지킴이 활동 등) |  |  |  |  |  |  |  |  |  |  |
| **환경 보호** (예: 에너지 절감사업 등) |  |  |  |  |  |  |  |  |  |  |
| **응급재난구호 지원** |  |  |  |  |  |  |  |  |  |  |

6. 다음은 **제약회사**의 사회기여 및 공헌활동 사례입니다. 각 활동별로 귀하가 생각하시는 **사회적 기대효과의 크기**를 1~10점에서 선택해 주십시오. (10점으로 갈수록 기대효과가 큼)

| 사회기여 및 공헌활동 | 1 | 2 | 3 | 4 | 5 | 6 | 7 | 8 | 9 | 10 |
| --- | --- | --- | --- | --- | --- | --- | --- | --- | --- | --- |
| **국민건강 증진** | | | | | | | | | | |
| 미치료 영역에서의 혁신적인 의약품 개발 |  |  |  |  |  |  |  |  |  |  |
| 신약개발 연구 지원 (예: 연구개발비 투자, 산학 공동연구 지원 등) |  |  |  |  |  |  |  |  |  |  |
| 취약계층 환자를 위해 자사 제품을 무료 혹은 저가로 제공 |  |  |  |  |  |  |  |  |  |  |
| 환자의 치료효과 향상을 위해 의약품 외 지원 활동(예: 환자대상 공개강좌, 당뇨환자에게 운동프로그램 지원 등) |  |  |  |  |  |  |  |  |  |  |
| 일반인 대상 질병 인식 개선활동 (예: 에이즈 캠페인, 정신건강 바로 알기 캠페인, 금연 교육 등) |  |  |  |  |  |  |  |  |  |  |
| 최신 의학 정보 제공 |  |  |  |  |  |  |  |  |  |  |
| **내부직원의 업무 및 복지환경 개선** | | | | | | | | | | |
| 제약회사 직원(내부고객)의 업무 및 복지환경 증진을 통한 삶의 질 개선 |  |  |  |  |  |  |  |  |  |  |
| **소외계층 지원** | | | | | | | | | | |
| 제약회사 제품과 직접 관련 없는 지역사회 봉사활동 (예: 연탄배달, 독거노인 돌봄 지원, 등) |  |  |  |  |  |  |  |  |  |  |
| 교육 프로그램 운영 (예: 다문화가정 학습 지원, 심리치유 프로그램), 장학금 지원 |  |  |  |  |  |  |  |  |  |  |
| **사회발전** |  |  |  |  |  |  |  |  |  |  |
| 제약회사의 고용 증진을 통한 일자리 증가 |  |  |  |  |  |  |  |  |  |  |
| 제약회사 제품과 직접 관련 없는 사회적 이슈 개선활동 (예: 장애인 인식 개선, 문화재지킴이 활동 등) |  |  |  |  |  |  |  |  |  |  |
| **환경 보호** (예: 에너지 절감사업 등) |  |  |  |  |  |  |  |  |  |  |
| **응급재난구호 지원** |  |  |  |  |  |  |  |  |  |  |
